# Unveiling the Potential Distribution of the Highly Threatened Madeira Pipistrelle (*Pipistrellus maderensis*): Do Different Evolutionary Significant Units Exist?

**DOI:** 10.3390/biology12070998

**Published:** 2023-07-13

**Authors:** Sérgio Teixeira, Sonia Smeraldo, Danilo Russo

**Affiliations:** 1 Faculty of Life Sciences (FCV), Universidade da Madeira, Campus da Penteada, 9000-082 Funchal, Madeira, Portugal; steixeira@staff.uma.pt; 2 Laboratory of Animal Ecology and Evolution (AnEcoEvo), Dipartimento di Agraria, Università degli Studi di Napoli Federico II, Via Università, 100, Portici, 80055 Naples, Italy; 3 Istituto Zooprofilattico Sperimentale del Mezzogiorno, Via della Salute, 2, Portici, 80055 Naples, Italy; sonia5.6.89@hotmail.it

**Keywords:** ecological niche models, endemic species, islands, Macaronesia, phylogenetic history, *Pipistrellus maderensis*, species distribution models

## Abstract

**Simple Summary:**

Island ecosystems, while hosting a significant proportion of global biodiversity and unique species, are exceptionally vulnerable. The considerable distances between islands and the mainland, coupled with the diverse environmental conditions, have had a profound impact on the evolutionary history of species. This study focuses on the Madeira, Azores, and Canary archipelagos, situated in the Macaronesia biogeographical region. Among the endangered endemic pipistrelle species, the Madeira pipistrelle (*Pipistrellus maderensis*) stands out. To compare the populations inhabiting these three archipelagos, we conducted genetic and ecological niche analyses. Our analyses revealed substantial differences in both DNA composition and habitat preferences among the populations. This research serves as a crucial starting point for future investigations, which aim to integrate ecological, morphological, genetic, and behavioural characteristics. By deepening our understanding of the distinctiveness of these populations and unravelling their evolutionary history, we can effectively address the conservation requirements of the Madeira pipistrelle. Consequently, we can ensure the long-term preservation of these remarkable bat populations and their vital ecological roles.

**Abstract:**

The isolation of islands has played a significant role in shaping the unique evolutionary histories of many species of flora and fauna, including bats. One notable example is the Madeira pipistrelle (*Pipistrellus maderensis*), which inhabits the Macaronesian archipelagos of the Azores, Madeira, and the Canary Islands. Despite the high biogeographic and conservation importance of this species, there is limited information on its ecology and evolutionary history across different archipelagos. In our study, we employed species distribution models (SDMs) to identify suitable habitats for the Madeira pipistrelle and determine the environmental factors influencing its distribution. Additionally, we conducted molecular comparisons using mitochondrial DNA data from various Macaronesian islands. Molecular analyses provided compelling evidence for the presence of distinct Evolutionary Significant Units on the different archipelagos. We identified distinct haplotypes in the populations of Madeira and the Canary Islands, with a genetic distance ranging from a minimum of 2.4% to a maximum of 3.3% between samples from different archipelagos. In support of this, SDMs highlighted relevant dissimilarities between the environmental requirements of the populations of the three archipelagos, particularly the climatic niche. Our research demonstrates that deeper investigations that combine ecological, morphological, and genetic areas are necessary to implement tailored conservation strategies.

## 1. Introduction

Islands represent unique geographic systems that harbour many endemic plant and animal species. Isolation, along with complex biogeographic processes, has resulted in the evolution of distinct lineages that are often found nowhere else on Earth [1]. Island biodiversity, therefore, represents a significant proportion of global biodiversity and is of exceptional conservation value.

Despite their ecological importance, island ecosystems are among the most threatened in the world. They are highly vulnerable to anthropogenic pressures such as overexploitation, habitat loss, invasive species, and climate change, making them hotspots for biodiversity loss [2]. Moreover, insular populations are often more vulnerable to extinction than their mainland counterparts due to their restricted geographic range [3], smaller sizes and densities [4], lower genetic diversity [3,5], and isolation [6]. 

Due to their exceptional ability to cover long distances in flight, bats play a crucial role as island colonisers and constitute one of the most diverse groups of mammals inhabiting such geographic systems [7]. Therefore, compared to the total number of mammal species, bats show greater proportions of island-dwelling, island-endemic, and single-island-endemic species [7]. 

Islands evoke a profound influence on the evolutionary trajectory of organisms, giving rise to distinctive adaptations, some of which exhibit remarkable conspicuousness (e.g., flightlessness in insular birds; [8]). While the adaptations in insular bats may be less pronounced, they exhibit intriguing behavioural modifications. For instance, these bats may display daytime activity, which can be best understood as a predator-release response [9,10,11]. Moreover, species that display significant behavioural specialisations on the mainland may demonstrate contrasting patterns when found on islands. A notable illustration of this phenomenon is observed in the long-fingered bat, *Myotis capaccinii*. While this species typically engages in trawling behaviour, skimming prey from the water surface on the mainland (e.g., [12,13]), it has adapted its hunting strategy on Zakynthos, a Greek island in the Ionian Sea. Due to the absence of suitable habitat, the bat has resorted to hunting prey on the wing within woodland habitats instead [14]. Finally, some peculiar patterns observed in the echolocation [15] and social calls [16] of insular bats can be attributed to isolation-driven processes. It is in this context that insular systems truly emerge as extraordinary natural laboratories, providing unparalleled opportunities to unravel the intricate mechanisms underlying the process of evolution in bats. 

In many cases, insular bat populations are, therefore, of especially high conservation value due to their unique evolutionary history and genetic diversity. Moreover, the decline of bat species residing on islands, encompassing those with insect-, nectar-, and fruit-based diets, engenders special apprehension due to the potential loss of vital ecosystem services (e.g., [17]). Islands, characterised by their isolation from the mainland, often exhibit diminished biodiversity and ecological imbalances [1], whereby bats assume a particularly pivotal role. These mammals play a crucial role in the pollination and dispersal biology of many plant species (e.g., [18]). Furthermore, the role of bats as effective agents in controlling insects that pose threats to agricultural crops, forests, and human well-being is well established based on studies conducted on the mainland (e.g., [19]). However, despite this recognition, the significance of this pest control role in island bat populations has been largely overlooked in existing research.

Bats exhibit exceptional sensitivity to anthropogenic pressures, with island populations, known for their delicate nature and small size, being particularly vulnerable. The encroachment of urbanisation, expansion of farmland and the associated spread of pesticides, the occurrence of wildfires, the effects of climate change, the proliferation of invasive species, and the direct persecution or even hunting of bats for sustenance collectively pose significant threats to insular bat populations worldwide [7,20,21]. Amidst this disconcerting scenario, endemic island bats emerge as a particularly precarious group, characterised by their high-risk status and inadequate research attention [20]. This insufficient research focus on insular bats has resulted in a disregard for the substantial risks they face from human activities, sometimes leading to a severe underestimation of their critical conservation status [22,23]. Moreover, this lack of attention has caused insufficient [24] or delayed [25] implementation of crucial conservation measures, ultimately pushing certain bat species to the brink of extinction and rendering their demise practically inevitable [26].

The plight of bats on islands has been predominantly recognised in the case of flying foxes. These species have been extensively hunted for consumption on various islands or targeted for their perceived contribution to crop depletion (e.g., [27,28,29,30,31]). Due to evolutionary factors and recent human-induced extinctions of other native dispersers, flying foxes frequently become solitary pollinators or dispersers on islands [32,33,34]. Thus, they act as keystone species that are vital for the sustainability of both indigenous or endemic island flora and the plants that are consumed or traded by local communities [35,36,37,38]. The loss of these species can trigger a chain reaction of extinction, resulting in catastrophic ecological, social, and economic consequences [30,32,39]. 

The decline of non-pteropodid bats on islands has received far less attention. These species are often endemic (e.g., [40]) or confined to one or a few islands (e.g., [41]), and their populations are typically small [23]. Similar to insular flying foxes, these bats too likely play a critical role in maintaining the balance of island ecosystems, especially by feeding on insect pests and other invertebrates. Therefore, the disappearance of these species might have a ripple effect on the entire island ecosystem, resulting in an increase in pests and potential crop damage and likely affecting the survival of other indigenous species. Given their limited distribution and small populations, conservation efforts for these bats are crucial to secure their future and ensure the ecological resilience of island habitats.

However, despite their ecological and conservation significance, insular “microbats” are frequently overlooked in conservation efforts, and as a result, many of these populations are facing imminent threats of extinction. One striking example is the Sardinian long-eared bat (*Plecotus sardus*), an endemic species found only in Sardinia. This bat is currently endangered due to various factors, including disturbances to its roosts and the impacts of climate change. Climate-related factors such as severe summer wildfires, alterations in precipitation patterns, and heat waves have all contributed to the bat’s decline [23]. These detrimental influences have led to a staggering population crash of over 63% between the years 2003 and 2020. 

While long-eared bats are specialised for specific habitats and prey, making them intrinsically vulnerable [42], pipistrelle bats, on the other hand, are typically regarded as adaptable generalists capable of adjusting to various environmental conditions and opportunistically feeding on a wide range of prey (e.g., [43,44]). However, this adaptability may not always hold true for insular pipistrelles. These bat species residing on islands may face a higher risk of endangerment and ultimately extinction. For instance, the European population of *Pipistrellus hanaki* is limited to a small area on Crete and the nearby Gavdos Island [45]. The species is threatened by the loss of roosts due to demolition and renovation works (P. Georgiakakis, pers. comm.). Additionally, tree roosts are endangered by deforestation for firewood, the expansion of agricultural areas at the expense of natural habitats, the replacement of ancient olive trees, urban and commercial development, wildfires, and the detrimental effects of overgrazing [45]. The construction of wind turbines also poses a significant threat to this species (P. Georgiakakis, pers. comm.).

An even more alarming example is provided by the Christmas Island pipistrelle (*Pipistrellus murrayi*), which was endemic to Christmas Island and experienced a severe decline first detected in the 1990s [46]. The exact cause of the decline remains unknown but is attributed to invasive species and possibly disease [47]. Despite being listed as Endangered in 2001, the species continued to decline rapidly. Urgent calls for conservation action, including captive breeding, were made, but the response was delayed [25]. In 2009, when only a few individuals remained, efforts to establish a captive breeding programme were initiated, but it was too late: by August 2009, the last pipistrelle could not be captured, and the species was declared extinct [25,26]. The delay in implementing conservation measures contributed to the species’ ultimate demise.

Significant events like the disappearance of the Christmas Island pipistrelle have captured the attention of researchers worldwide, leading to a focus on insular bats [20,40,48,49,50,51]. This attention has spurred numerous studies on the genetic diversity of pipistrelle complexes in the Mediterranean and Macaronesia, particularly among widespread pipistrelle species like *Pipistrellus pipistrellus* and *Pipistrellus kuhlii* [48,52,53,54,55,56].

Mitochondrial DNA tools have played a vital role in assessing the taxonomic significance of this diversity within the various species complexes, leading to the identification of several cryptic species. For instance, the use of these tools revealed the presence of *Pipistrellus pygmaeus* as a distinct species in mainland Europe [57] and the recognition of the island species Hanak’s pipistrelle, *Pipistrellus hanaki* [58]. However, recent phylogenetic and morphological studies have emphasised that the taxonomy of pipistrelles on certain islands still requires clarification. This indicates that the diversity of Palearctic pipistrelles in various regions remains to be fully discovered and understood [48,53,59]. These findings underscore the urgency of assessing the taxonomic status of several island pipistrelles in Europe, particularly within the Mediterranean and Macaronesian biogeographical regions. These regions are home to numerous islands with small populations of insular bats [60,61,62,63].

*Pipistrellus maderensis* represents one of the endangered endemic pipistrelle species found in insular habitats. This small insectivorous bat inhabits the Atlantic islands of Madeira and Porto Santo, the Azores, and the Canary Islands [60,61,64,65,66,67]. While we have a good understanding of its distribution in Madeira [50,61,64] and the Canary Islands [68,69], there are significant gaps in our knowledge regarding its presence in the Azores (but see [60,70,71]. 

The population of *P. maderensis*, like other pipistrelles, is facing significant declines primarily due to factors such as habitat destruction, forest fires, urban development, and persecution. On Madeira Island, areas where the species was once abundant in the late 1990s now show little to no activity or only sparse occurrences (S. Teixeira, personal observation). Similarly, many of the larger known roosting sites have been destroyed by major fires in 2010, 2012, and 2016 or through direct actions targeting bat roosts, as these mammals are considered pests by many of the island’s inhabitants.

Although some ecological data have been collected by various researchers [60,61,64], the lack of comprehensive information regarding species distribution and ecology presents significant challenges for conservation efforts. Therefore, it is crucial to comprehend the suitability of its habitat and potential distribution to identify areas requiring conservation management and to prioritise future research endeavours. Additionally, the considerable geographic distance between the Macaronesian archipelagos where this species is found suggests limited gene flow, indicating the potential existence of distinct Evolutionary Significant Units (ESU).

To address this crucial knowledge gap, we combined molecular analysis with the potential distribution modelling of *Pipistrellus maderensis* across Macaronesia. We identified suitable habitats by employing a species distribution model (SDM) and determined the environmental factors influencing the species distribution. Moreover, we leveraged available data to examine and compare mitochondrial DNA information from various Macaronesian islands to elucidate the presence of different ESU. We hypothesise that both modelling and mitochondrial analyses will support the existence of separate ESUs in the different Macaronesian insular systems. Our analysis sought to provide essential insights into the ecological requirements of the species, identify potential management units, and pinpoint areas worthy of future field investigations.

## 2. Materials and Methods 

### 2.1. Study Area

**Figure 1:**
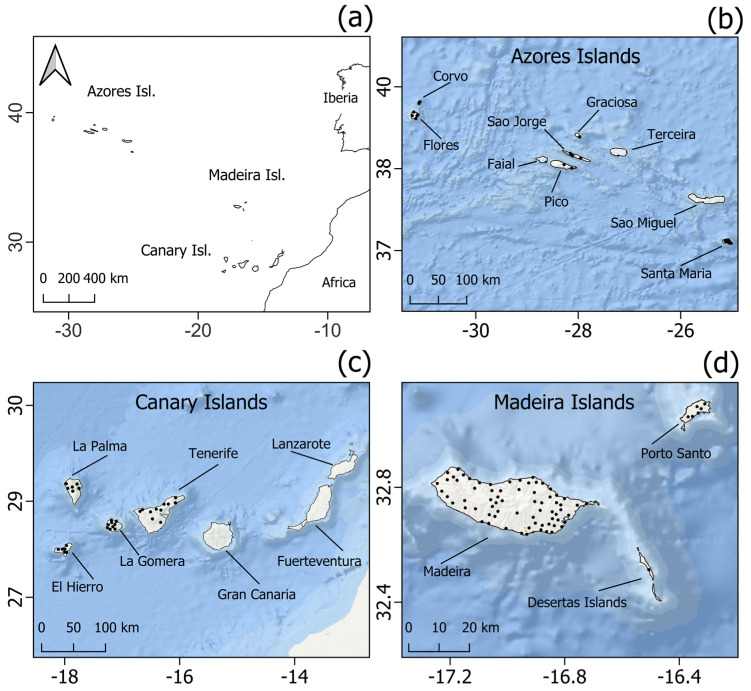
Geographical location of the three Macaronesian archipelagos included in this study in relation to Africa and Europe (**a**). Presence records (black dots) of *P. maderensis* in the Azores (**b**), Canary (**c**), and Madeira (**d**) Islands.

Our study was conducted in the Macaronesian biogeographic region (Figure 1a), specifically in the archipelagos of the Azores, Madeira, and the Canaries. These archipelagos are in the North Atlantic Ocean, off the coasts of Europe and Africa, spanning between 27° and 39° N latitude and between 13° and 31° W longitude. Each archipelago consists of several oceanic islands, which were formed by seamounts on the ocean floor. Despite their geographical separation, these islands share common environmental characteristics, including volcanic origins, diverse landscapes, and climates [72,73,74,75,76].

The Macaronesian islands boast remarkable biodiversity hotspots, owing to the presence of large calderas, rugged mountains and cliffs, expansive valleys, and sheltered bays [50,65,77,78]. In terms of climate, the Macaronesian islands exhibit a wide range of variations. The Azores and Madeira experience maritime temperate, Mediterranean, and subtropical climates. Some of the Canary Islands have Mediterranean and subtropical climates, while others, particularly Lanzarote and Fuerteventura, have more arid climates due to their geologically older nature. The southernmost islands exhibit a tropical climate. These climatic differences are influenced by factors such as the Azores’ anticyclone, varying latitudes and ages, and the geomorphology of the islands. Additionally, the distance to the coast and altitude also contribute significantly to the local climate [79].

In the Madeira Archipelago, the land is predominantly covered by forests and semi-natural areas, with the subtropical laurel forest being the dominant native vegetation. A similar situation is observed in the Canary Islands and the Azores, where forests and semi-natural areas account for approximately three-quarters of the territory. However, human activities have caused profound alterations in the land cover of these islands, resulting in the dominance of pastures and planted forests.

### 2.2. Phylogenetic Analysis

To compare populations of *Pipistrellus maderensis* in the Macaronesian archipelagos of Madeira, the Azores, and the Canary Islands, sequences of the *P. maderensis* partial cytochrome b (cyt b) gene were taken from GenBank using BLASTN. A total of 15 *P. maderensis* partial cyt b sequences were selected and downloaded as complete sequence FASTA files. The selected sequences included five haplotypes from Madeira Island in the Madeira archipelago (Accession numbers KC520770–KC520774) and 10 haplotypes from the Canary Islands (Accession numbers AJ426610–AJ426618 and AJ426632) from all four islands where the species occurs in this archipelago (Tenerife, La Palma, La Gomera, and El Hierro). There were no available sequences from the Azores archipelago; therefore, our analysis only included the sequences from Madeira and the Canary Islands. Given the ongoing challenge of resolving the *P. kuhlii* complex lineages in Southern Europe, Northern Africa, and the Canary Islands [48,55,56], and the confirmed presence of Kuhl’s pipistrelle in the Canary Islands, where hybridisation with Madeira pipistrelles has been observed [69], we made the decision to include a sequence of *P. pipistrellus* from Iberia (Accession number EU360671) in our dataset. This sequence will serve as the outgroup for our analysis. The used nucleotide database is non-redundant, so identical haplotype sequences were merged into one entry and excluded from the final dataset, resulting in 12 cyt b sequences with a total of 478 nucleotide positions (Table 1). The obtained GenBank final dataset FASTA file was imported into the software MEGA v.11 013 [80] and aligned with the MUSCLE algorithm using the default parameters. Substitution patterns and rates were estimated under the Tamura-Nei model [81]. A discrete Gamma distribution was used to model evolutionary rate differences among sites. We also estimated the number of base substitutions per site to obtain the Average Evolutionary Divergence over all sequence pairs. Considering the small dataset (*n* = 12), we used the maximum likelihood method (ML) for the phylogenetic analysis and tested 24 different evolutionary models, of which we selected the best-performing model using the Bayesian information criterion (BIC) and the corrected Akaike information criterion (AICc). For estimating ML values, a tree topology was automatically computed. Calculation of evolutionary distances was performed using MEGA v.11 013 [80] based on the Tamura-Nei model [81]. Trees were visualised using FigTree v.1.3.1 [82].

### 2.3. Occurrence Records

Presence records of *Pipistrellus maderensis* were collected from various published sources across all three archipelagos, resulting in a total of 613 occurrences across 13 different islands (Table 2). We aimed to encompass the entire known distribution of the species to capture a broader range of suitable conditions in which the species may occur. To avoid redundancy, the dataset was thinned to a 1 km resolution to align with the grids of the environmental layers. Spatially autocorrelated points were removed from the dataset using the Spatially Rarefy Occurrence Data tool of SDMtoolbox v. 2.4 [84] in ArcGIS v. 10.8 [85]. The final dataset consisted of 28, 41, and 92 occurrences for the Azores, Canaries, and Madeira archipelagos, respectively, totalling 161 presence records (Figure 1b–d).

### 2.4. Environmental Predictors

To analyse the current potential distribution of *P. maderensis* in the Macaronesian region, we considered a set of environmental predictors. These predictors included elevation, 19 bioclimatic variables derived from the Worldclim database ver. 2.1 (https://www.worldclim.org/data/worldclim21.html, accessed on 15 December 2022 [89]), land-use categories extracted from the Corine Land Cover database (CLC ver. 2018), artificial illumination (layer annual 2019) provided by the National Oceanic and Atmospheric Administration (NOAA) (https://www.lightpollutionmap.info/, accessed on 9 January 2023), and hydrographic elements obtained from the Overpass Turbo platform (https://overpass-turbo.eu/, accessed on 23 November 2022).

Elevation and the bioclimatic variables, which represent monthly mean temperature and precipitation values (or minimum and maximum temperature when available), were downloaded at a resolution of 30 s (~1 km^2^ at the equator). The artificial illumination and CLC layer had a resolution of 100 × 100 m. Within the CLC, we selected only those land cover categories considered significant in defining suitable habitats for *P. maderensis*. The hydrographic elements included rivers, artificial canals, ponds, and lakes. To ensure that all environmental predictors were in a continuous, ratio-scaled raster format, we calculated the Euclidean distances for the hydrographic elements and land cover categories. Additionally, all predictors were rasterised at a resolution of approximately 1 km using the resample tool in ArcGIS (ver. 10.8). The layers were cropped to include the Macaronesia region.

To account for pairwise correlations between predictors, the final set of variables was sub-selected based on Pearson’s correlation coefficient |r| < 0.7 [90,91]. Within each group of highly correlated predictors, we chose the variables that were potentially useful in predicting suitable habitats for *P. maderensis*, guided by expert knowledge of the species’ ecology. As a result, the final list of the most relevant variables used to model *P. maderensis*’s potential distribution in the Macaronesian archipelago includes the mean diurnal range (BIO2), minimum temperature of the coldest month (BIO6), annual precipitation (BIO12), Euclidean distance from mixed agricultural lands and natural vegetation, Euclidean distance from hydrographic elements, and artificial illumination.

### 2.5. Model Development and Analysis of Environmental Matching

To generate projections of the potential distribution of *P. maderensis* in the Macaronesian region, we employed an ensemble modelling approach [92] using the biomod2 package [93] in the R platform (version 4.2.1) R Foundation for Statistical Computing Platform. The ensemble modelling approach combines predictions from independent models using different techniques. In this study, we utilised three commonly used statistical modelling techniques: generalised boosting models or boosted regression trees (GBM, [94]), random forests (RF, [95]), and generalised linear models (GLM).

Initially, we calibrated our model using all records from the known species distribution in the Macaronesian region. Since only presence information was available, we performed five runs of pseudo-absence (PA) generation using the Surface Range Envelope (SRE) method. The SRE method randomly selects pseudo-absences within the range of environmental conditions that differ from those of the presence points. This pseudo-absence selection method is particularly suitable when the entire climatic niche of the species has been sampled, which is the case in our data.

We conducted five runs to optimise model quality for regression techniques, with pseudo-absences covering approximately 2% of the background area [96]. As for GBM and RF models, it is suggested to select the same amount of PA as available presences, while for GLM a larger number of PA (10,000) [96], we decided to select for each run a number of PA equal to three times the number of presences in accordance with the extent of the study area.

To evaluate the predictive performance of the models, we randomly split the occurrence dataset into a 70% sample for model calibration and the remaining 30% for model validation. We repeated this procedure five times and averaged the results. The predictive performance of species distribution models (SDMs) was assessed using the area under the receiver operating characteristic curve (AUC; [97]) and the true skill statistic (TSS; [98]). AUC values were used to categorise prediction accuracy as excellent (AUC > 0.90), good (0.80 < AUC < 0.90), fair (0.70 < AUC < 0.80), or poor (AUC < 0.60). TSS values categorise prediction accuracy as excellent (TSS > 0.75), good (0.40 < TSS < 0.75), or poor (TSS < 0.40). Only models with AUC values ≥ 0.70 were considered for subsequent analyses [99].

Ensemble models were created by calculating a weighted average of the individual model predictions, with the AUC scores serving as weights [100]. The models were then projected over the study area, and the relative importance of variables was determined using permutation tests on the ensemble model. The final potential distribution was obtained by averaging the projections from the 25 replicated ensemble models generated through the subsampling procedure. The resulting map was binarised into presence-absence values using a threshold that maximised both sensitivity (the percentage of correctly predicted presence) and specificity (the percentage of correctly predicted absence) [101]. The threshold selection mentioned is widely used and considered accurate in various studies [102,103,104,105].

After generating the SDM calibrated on all records, we assessed the reliability of the predicted potential distribution by testing the degree of matching between the environmental conditions of the three archipelagos included in the study area. Considering the distance between the archipelagos (ranging from a minimum of 414 Km between Madeira and the Canary Islands to a maximum of 1112 Km between the Azores and the Canary Islands) and the diverse climatic and environmental conditions present, projecting model predictions to a novel space may result in a portion of the environment covering additional conditions outside the calibration range. These non-analogue conditions and extrapolations beyond the calibration range of the model need to be evaluated [106,107,108,109,110].

To assess spatial extrapolation and identify areas where environmental conditions are outside the range of conditions contained in the calibration area, we employed the Multivariate Environmental Similarity Surface (MESS) approach. This approach analyses spatial extrapolation by extracting environmental values covered by presence-only records and estimating areas with conditions beyond the range of the calibration area [111].

We calculated the degree of matching between temperature conditions in the calibration and projection ranges of the species using the dsmextra package [112], an R implementation of the Extrapolation Detection (ExDet) tool proposed by Mesgaran et al. [113]. The ExDet tool allows for quantitative, spatially explicit assessments of univariate and combinatorial extrapolation using Euclidean and Mahalanobis distances [112]. ExDet values ranging from 0 to 1 indicate temperature conditions analogous to the calibration range (environmental matches), while values >1 or <0 indicate novel temperature conditions that are non-analogous to those experienced in the calibration range, potentially causing model extrapolation. Values < 0 indicate out-of-range values for any given covariate (univariate extrapolation), while values > 1 indicate novel combinations of values within the univariate range of the reference covariates (combinatorial extrapolation).

Finally, in response to the outcome of the MESS analysis (see Section 3.2), we developed three separate SDMs, one for each archipelago, by calibrating the models on records from the Azores, Canaries, and Madeira individually. We applied the same modelling approach as the general model. For each archipelago, we developed SDMs using different combinations of uncorrelated predictors and selected the model with the highest performance in terms of AUC, considering the predictors that contributed significantly to determining habitat suitability for *P. maderensis*.

## 3. Results

### 3.1. Genetic Analysis

**Figure 2:**
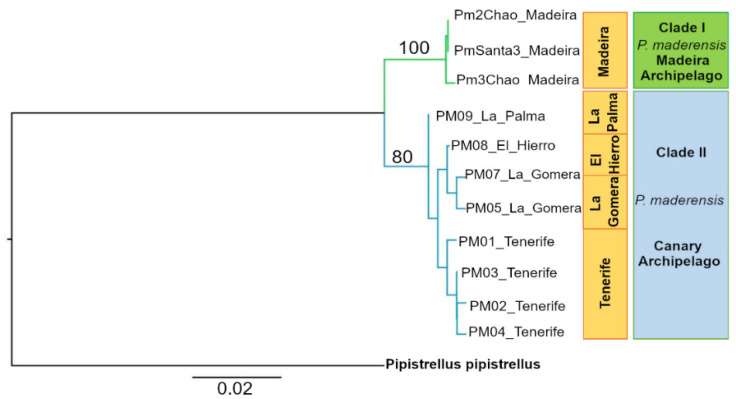
The maximum likelihood tree depicts the genetic relationships among cytochrome b haplotypes of *Pipistrellus maderensis* from Madeira and the Canary Islands, based on a 478 bp fragment. Bootstrap values indicating the support for each clade are presented above the respective nodes. To establish the root of the tree, *Pipistrellus pipistrellus* from Northern Iberia was used as an outgroup species.

Distinct haplotypes were observed in the populations of Madeira and the Canary Islands, with no shared sequences of cyt b between the two archipelagos (Figure 2). The phylogenetic analysis clearly differentiated the populations from both archipelagos (Figure 2). The Tamura-Nei distance revealed that the minimum genetic distance between the Madeira and Canary archipelagos was 2.4%. This distance was specifically observed between samples from Madeira Island (Pm2Chão from Chão da Ribeira, Porto Moniz) and La Palma Island (PM#09 from Mirca, Santa Cruz). The maximum distance of 3.3% was found between Madeira Island (Pm2Chão from Chão da Ribeira, Porto Moniz) and Tenerife Island (PM#01 from Cueva Fea de Arico and La Orotava).

The Tamura-Nei model analysis of evolutionary divergence within each archipelago yielded different results regarding the genetic diversity of each insular population. In Madeira, the sampled bats exhibited an average genetic distance of 0.001%, while within the Canary archipelago, the samples showed an average distance of 1.7% (Figure 2), demonstrating that the genetic diversity of *P. maderensis* in the Canary archipelago is significantly higher than that of the population in Madeira Island. The estimated average evolutionary divergence across all sequence pairs was 3%.

Among the models evaluated for the evolutionary history of *P. maderensis* in Madeira and the Canary Islands, the Tamura-Nei model performed the best, with a Bayesian BIC score of 2267.024 and an AICc of 2096.229. The maximum likelihood value was ln (L) = −1025.442. The estimated transition/transversion bias (R) was 6.716. In the ML tree (Figure 2), the Madeira haplotypes (Clade I) were strongly supported by bootstrap values (BS = 100), while the Canary Islands node had a lower bootstrap value (BS = 80). Nevertheless, bootstrap values remain high, and the node is well supported.

### 3.2. Macaronesian Model and MESS Analysis

**Figure 3:**
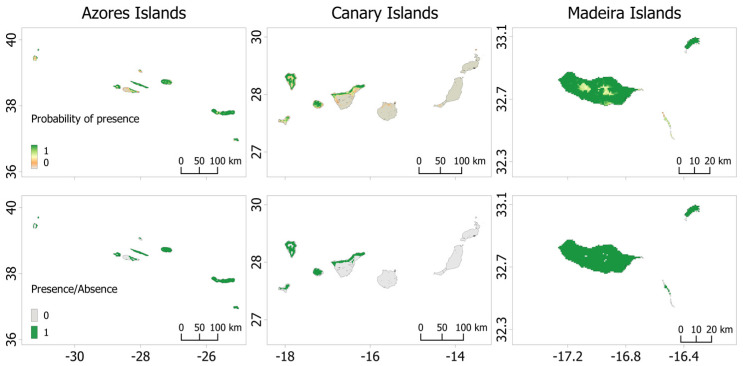
*Pipistrellus maderensis* Species Distribution Models for the Azores, Canary, and Madeira archipelagos. **Top**: logistic map; **Below**: binary map. Scales show the probability of presence, ranging from 0 to 1.

Our species distribution model (SDM), calibrated using all the presence records and projected to the Macaronesian archipelagos, demonstrated robust predictive performance for *P. maderensis*, as evidenced by high values of the area under the receiver operating characteristic curve (AUC > 0.9) and true skill statistic (TSS > 0.8). Among the modelling algorithms used, random forest exhibited the highest performance, with an AUC of 0.97 (Table 3). However, the resulting model exhibited an unrealistic potential distribution for *P. maderensis*, particularly in Madeira and the Azores, where the model overestimated the extent of suitable habitat compared to the known current distribution of the species (Figure 2 and Figure 3). As an example, the binary map of Madeira erroneously predicts the presence of *P. maderensis* throughout the entire island, including areas where it is known that the species does not exist due to unsuitable environmental conditions. Moreover, analysis of the response curves for the environmental variables indicated that their influence on the probability of species presence was relatively weak, with the mean diurnal range of temperature demonstrating the highest contribution to the model (Figures S1 and S2).

**Figure 4:**
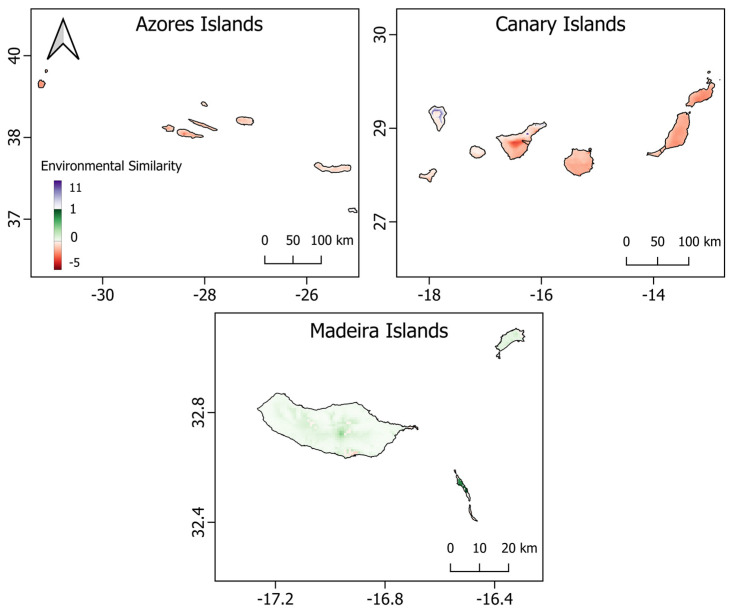
MESS map of *Pipistrellus maderensis* in the projection areas. The maps show the similarity of the environmental variables between training and projection data. Environmental similarity values ranged from negative (red) to positive (blue) to zero (white). Areas in red or close to red have one or more environmental variables whose values fall outside the range observed in the training area (Madeira), while areas in blue or close to blue indicate novel combinations of values encountered within the univariate range of the reference covariates. Green areas (comprised between 0 and 1) show environmental conditions analogous to the calibration range (environmental matches).

These findings were supported by the results of the Multivariate Environmental Similarity Surface (MESS) analysis, which revealed significant dissimilarity in environmental conditions between Madeira and most of the surface area of the Azores and Canary Islands, both in terms of univariate and combinatorial extrapolation (Figure 4). Specifically, the analysis detected and quantified the degree of dissimilarity for points that fell outside the univariate range or formed new combinations of covariates. Our results indicated that approximately 90% of the surface area of the Azores and Canary Islands exhibited environmental conditions outside the range of the considered variables for Madeira. The mean diurnal range of temperature and annual precipitation were the most differentiating predictors among the three archipelagos, contributing to 53% and 36% of the total environmental dissimilarity, respectively (Table 4). The distance from agricultural lands and hydrographic elements were the only two predictors that contributed to dissimilarity in terms of combinatorial extrapolation. These results underscored the limited transferability of the model calibrated on Madeira to the environmental conditions of the other two archipelagos, highlighting the necessity of separately projecting the potentially suitable habitat of *P. maderensis* for each archipelago.

### 3.3. Archipelago-Specific Models

The species distribution models (SDMs) developed for each archipelago demonstrated good predictive performances, with TSS values > 0.7 and AUC values > 0.8 (Table 5). The three resulting SDMs incorporated slightly different sets of environmental predictors (Table 6) and provided a more realistic potential distribution of *P. maderensis* across the Macaronesian Islands compared to the model calibrated on all islands.

**Figure 5:**
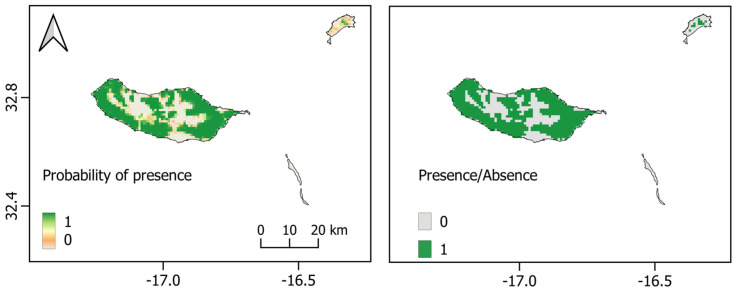
*Pipistrellus maderensis* Species Distribution Models for Madeira and Porto Santo. **Left**: logistic map; **right**: binary map. Scales show the probability of presence, ranging from 0 to 1.

In the case of Madeira and Porto Santo, our models accurately predicted suitable habitats for *P. maderensis* (Figure 5). The annual mean precipitation emerged as the most influential variable in predicting species occurrence. The smoothed response curves indicated a strong dependence on areas with annual precipitation of approximately 700 mm and a mean diurnal range between 4.5 and 5.5 degrees. Additionally, the model predicted a decrease in suitable habitats with increasing distance from hydrographic elements and agricultural lands, while lower levels of artificial illumination were likely to favour the species’ occurrence (Figure S3). However, these latter predictors exhibited a lower contribution to the model for the Madeira archipelago.

**Figure 6:**
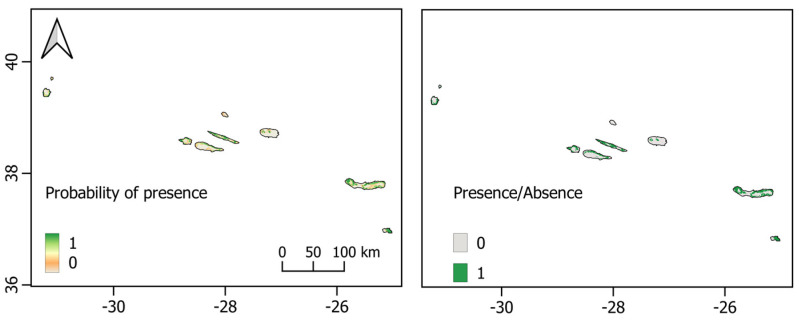
*Pipistrellus maderensis* Species Distribution Models in the Azores Archipelago. **Left**: logistic map; **right**: binary map. Scales show the probability of presence, ranging from 0 to 1.

The model projected to the Azores Islands generally aligned with the known species distribution on most islands, except for San Miguel and Faial, where it predicted numerous suitable areas with potentially favourable climatic conditions despite the species not yet being detected there (Figure 6). The minimum temperature of the coldest month emerged as the most important variable, with a peak potential probability of occurrence for *P. maderensis* between 10 and 12 °C, followed by isothermality and distance from agricultural lands (Figure S4). Similar to Madeira, the model for the Azores archipelago indicated increased habitat suitability at shorter distances from hydrographic elements and agricultural lands, as well as in areas characterised by lower levels of artificial illumination.

**Figure 7:**
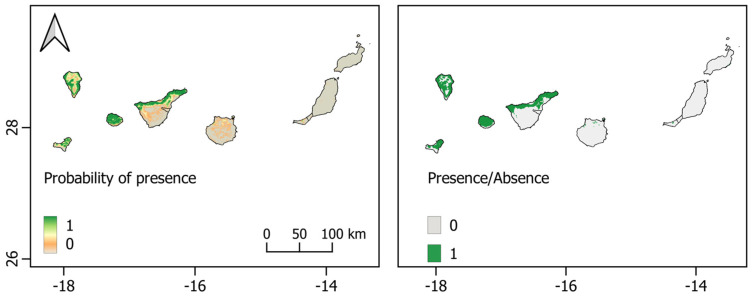
*Pipistrellus maderensis* Species Distribution Models in the Canary Archipelago. **Left**: logistic map; **right**: binary map. Scales show the probability of presence, ranging from 0 to 1.

In the case of the Canary Islands, the analysis of environmental variable contributions revealed that annual mean precipitation was the primary bioclimatic variable influencing model performance. Based on the model predictions, *P. maderensis* displayed a higher likelihood of occurrence in regions with precipitation ranging from 300 to 400 mm and a mean diurnal temperature range below 7 °C (Figure S5). Specifically, considering the different climatic conditions among the Canary Islands, the species distribution model (SDM) identified potentially suitable habitats in multiple locations along the north coast of Tenerife, as well as on the islands of La Gomera, La Palma, and El Hierro, characterised by the higher value of annual precipitations. Conversely, limited or no suitable environmental conditions were predicted on Gran Canaria, Lanzarote, and Fuerteventura islands (Figure 7), where the climate is more arid.

## 4. Discussion

Consistent with our hypothesis, both the modelling and mitochondrial analyses support the presence of distinct Evolutionary Significant Units (ESUs) in the various Macaronesian archipelagos. The phylogenetic analysis clearly demonstrates that the populations of *P. maderensis* in Madeira and the Canary Islands form separate clades. The substantial 3.3% evolutionary distance between these populations indicates their distinctiveness and confirms the existence of at least two different ESUs. These phylogenetic differences corroborate the findings of the MESS model, which indicates that each insular population occupies islands with notable environmental dissimilarities across the three archipelagos. As a result, it underscores the pressing need to investigate these populations further and implement conservation measures tailored to each specific archipelago.

The lower within-archipelago genetic diversity observed in Madeira, compared to the Canaries, is likely due to differences in the number of islands sampled in each archipelago and sites per island. *Pipistrellus maderensis* inhabits four islands (La Palma, El Hierro, La Gomera, and Tenerife) within the Canary Archipelago [68,69], while it is found on three islands in the Madeira Archipelago (Madeira, Porto Santo, and Deserta Grande) [60,61,64,65,114]. In the Canary Islands, 15 sites across all the islands were sampled and sequenced, whereas in Madeira, only haplotypes from two sites on Madeira Island were available. In Madeira, the *P. maderensis* population in Deserta Grande may have become extinct due to extensive habitat destruction over centuries, while the population in Porto Santo was very small in the early 2000s. Moreover, all the samples from Madeira are limited to the northwestern locality of Porto Moniz, resulting in a sparse representation of the genetic diversity on this island. Consequently, the analysis for the Madeira archipelago includes only one island and is still undersampled, leading to lower observed genetic diversity compared to the Canary Islands. Hence, it is crucial to conduct extensive sampling of *P. maderensis* throughout both Madeira Island and Porto Santo Island within the Madeira archipelago. This comprehensive sampling effort is essential to accurately assess the haplotypes present in the archipelago and to establish a comprehensive model of the evolutionary history of the Madeiran *P. maderensis* clade. Such an approach will provide robust evidence supporting its distinction as an Evolutionary Significant Unit (ESU) within the broader population of *P. maderensis* in the Macaronesian region.

The genetic characterisation of Azorean pipistrelle populations remains incomplete, which poses a significant obstacle to fully understanding their taxonomic and conservation status. The differences uncovered in this study underscore the importance of sampling the Azorean populations and expanding sampling efforts in the Madeira archipelago. Such endeavours are essential to accurately define the Evolutionary Significant Units (ESUs) of the Madeira pipistrelles and to refine our understanding of the genetic divergence among these three island groups. Moreover, conducting acoustic and morphological studies in each archipelago is crucial to complement and support these findings.

While some morphological studies have been conducted in Madeira and the Canary Islands [61,68], no comparative studies have been undertaken between these two insular populations. Furthermore, it is important to highlight that there is a lack of published studies focusing on the Azores. In the context of the Canary Islands, it becomes crucial to use morphological data during sample collection to prevent misidentification, as some specimens previously identified as *P. maderensis* from the Islands of Tenerife and La Palma were found to be *P. kuhlii* [69]. This underscores the necessity to deepen our understanding of the morphological distinctions between *P. maderensis* and *P. kuhlii* and to establish clear boundaries between intraspecific polymorphism and interspecific characteristics for these closely related species. Several studies have indicated the presence of the *P. kuhlii* "eastern lineage" in the Canary Islands [48,55,56,83], and there is evidence of hybridisation between *P. kuhlii* and *P. maderensis* in Tenerife and La Palma [69]. Therefore, it is crucial to conduct further research to enhance our knowledge of these species and their potential interactions in the Canary Islands.

Therefore, performing comprehensive acoustic and morphological investigations across the different archipelagos is vital to enhancing our understanding of the evolutionary history and taxonomic status of *P. maderensis* clades in these islands. Acoustic studies have primarily focused on the Madeira archipelago, where detailed investigations of *P. maderensis* echolocation and social calls have been conducted [16,61,64]. In contrast, limited acoustic samples were taken and analysed in the Azores [60], but a comprehensive characterisation of the echolocation acoustic parameters for the various pipistrelle populations in these islands has not been conducted. As a result, our knowledge regarding the echolocation patterns of the Canary populations of *P. maderensis* remains incomplete and requires further investigation. Further research is necessary to fill these knowledge gaps and provide a comprehensive understanding of the acoustic characteristics and variations among the different populations of *P. maderensis* across the Canary, Madeira, and Azores archipelagos.

Considering species distribution models, previous studies [115,116,117] have suggested the importance of focusing on intraspecific differentiation and potential niche divergences in species with allopatric populations. In this perspective, our findings show that archipelago-based SDMs demonstrate different responses of *P. maderensis* populations to environmental conditions, leading to different results compared to the SDM that considers the species in its entire range. This ecological niche dissimilarity is reflected by both the different sets of variables and the range of values of the individual predictors that determine the species suitable habitat in the three archipelagos. For instance, in Madeira and the Canary Islands, the range of annual mean precipitation with a high probability of species occurrence is 700 mm and 400 mm, respectively. Additionally, in the Azores Islands, the primary environmental predictor influencing the species’ habitat suitability is the minimum temperature of the coldest month, which, conversely, is not significant for modelling the potential distribution of the species in the other two archipelagos. Therefore, our modelling and genetic analysis results, as well as the test of model transferability (MESS analysis), support the hypothesis of the existence of different ESUs within the Macaronesian region. This highlights the importance of integrating intraspecific differentiation into SDMs (i.e., ESU-based models; [117]) to better understand the habitat suitability of different populations of *P. maderensis* and enhance the effectiveness of conservation plans. 

Regarding habitat suitability and the probability of the presence of *P. maderensis* across the Macaronesian Islands, the models have consistently supported the notion that the species is more widespread in the Madeira archipelago compared to other Macaronesian archipelagos [16,61,64,65,114]. However, despite the overall suitability of most areas on Madeira and Porto Santo islands, significant habitat loss and synanthropic pressures over centuries have led to severe population declines in coastal areas of Madeira Island and the entire Island of Porto Santo [60,61,64,65].

In the case of Desertas (the Madeira archipelago), the model indicates that two of the smaller islands still exhibit suitable conditions for *P. maderensis*, particularly Deserta Grande Island. However, the probability of occurrence for the species is relatively lower. It is worth noting that *P. maderensis* was known to inhabit Deserta Grande Island until the late 1990s. Nevertheless, extensive surveys conducted between 2014 and 2016 using ultrasound detectors did not detect any bats on the island. This suggests a potential local extinction of the small population. The destruction of plant cover caused by introduced goats, rabbits, and house mice, along with desertification and water scarcity on the island, likely contributed to the demise of this population.

## 5. Conclusions

In conclusion, our study highlights the genetic distinctiveness of the Madeira pipistrelle populations across the Macaronesian archipelagos as well as their specific ecological preferences identified through our modelling efforts. These findings support their classification as separate Evolutionary Significant Units (ESUs) and emphasise the significance of their uniqueness. Considering the ongoing challenges posed by urbanisation, the loss of suitable roosting sites, and the degradation or loss of foraging habitats, it is crucial to incorporate the distinctiveness of these bat populations into the development and implementation of tailored management strategies. Moreover, their unique identity should be appropriately acknowledged in future red-list assessments to ensure their conservation needs are adequately addressed.

Furthermore, our study has identified important research gaps that should be addressed in the future to gain a comprehensive understanding of the colonisation history and evolutionary trajectory of the Madeira pipistrelle across the diverse geographic settings it has inhabited. Filling these gaps will contribute to a more holistic evaluation of their evolutionary history and aid in formulating effective conservation measures for their long-term survival.

## Figures and Tables

**Table 1:** The cyt b sequences of *Pipistrellus maderensis* considered for the present study, their geographic origin, accession numbers, and corresponding references.

Archipelago	Island	Locality	Code/ Haplotype	Accession Number	Source
Canary					
	El Hierro	Frontera	PM#06	AJ426615	[69]
		Guarazoca	PM#08	AJ426617	[69]
	La Gomera	Los Acevinos	PM#06	AJ426615	[69]
		Agulo	PM#06	AJ426615	[69]
		Alajero	PM#06	AJ426615	[69]
		Tamargada	PM#05	AJ426614	[69]
		Juego de Bolas	PM#07	AJ426616	[69]
		Vallehermoso	PM#06	AJ426615	[69]
	La Palma	Mirca	PM#09	AJ426618	[69]
		Mirca	PM#11	AJ426632	[69]
		El Paso	PM#10	n.a.	[69]
		Los Sauces	PM#10	n.a.	[69]
	Tenerife	Cueva Fea de Arico	PM#01	AJ426610	[69]
		Cueva Fea de Arico	PM#03	AJ426612	[69]
		Cueva Fea de Arico	PM#04	AJ426613	[69]
		La Orotava	PM#01	AJ426610	[69]
		Almáciga	PM#02	AJ426611	[69]
		La Esperanza	PM#03	AJ426612	[69]
Madeira					
	Madeira	Sta. Porto Moniz	PmSanta1	KC520774	[83]
		Sta. Porto Moniz	PmSanta2	KC520773	[83]
		Sta. Porto Moniz	PmSanta3	KC520770	[83]
		Chão da Ribeira	Pm2Chao	KC520771	[83]
		Chão da Ribeira	Pm3Chao	KC520772	[83]

**Table 2:** Occurrences of *P. maderensis* and corresponding data sources used in SDM for this study, organised by archipelago and island.

Archipelago	Island	Number of Records	Source
Azores			
	Corvo	1	[60]
		2	[71]
	Flores	6	[60]
		8	[71]
		1	[62]
	Graciosa	1	[71]
	Pico	2	[71]
	Santa Maria	9	[60]
		15	[63]
		14	[70]
	São Jorge	5	[71]
Canary			
	El Hierro	3	[68]
		5	[69]
		3	[86]
	La Gomera	5	[68]
		6	[69]
		16	[86]
	La Palma	5	[68]
		3	[69]
		14	[86]
	Tenerife	5	[68]
		4	[69]
		1	[87]
		21	[86]
Madeira			
	Madeira	386	[61]
		65	[64]
		1	[88]
	Porto Santo	3	[60]
		3	[61]
	Deserta Grande	1	Silva (pers. obs.)

**Table 3:** The total model’s predictive performance for each modelling algorithm indicated by the mean and standard deviation (in brackets) of the AUC and TSS values.

Model	AUC (sd)	TSS (sd)
GBM	0.96 (0.02)	0.85 (0.05)
GLM	0.94 (0.02)	0.80 (0.04)
RF	0.97 (0.01)	0.84 (0.05)

**Table 4:** Percentage of the study area interested by univariate and combinatorial extrapolation, respectively, considering the influence of each environmental predictor separately based on the results of the MESS analysis.

Variable	Univariate Extrapolation (%)	Combinatorial Extrapolation (%)
BIO2	53	0
BIO12	36	0
Artificial illumination	0.6	0
BIO6	0.4	0
Euclidean distance from mixed agricultural lands	0.05	2.4
Euclidean distance from hydrographical elements	0.03	0.26

**Table 5:** The predictive performance resulted from the SDMs of each archipelago, indicated by the mean and standard deviation (in brackets) of the AUC and TSS values.

Model	AUC (sd)	TSS (sd)
Azores	0.87 (0.08)	0.72 (0.15)
Canary Islands	0.91 (0.06)	0.77 (0.13)
Madeira	0.88 (0.04)	0.71 (0.08)

**Table 6:** The important environmental predictors selected to develop the SDMs predict the occurrence of *Pipistrellus maderensis* in each archipelago.

Azores Archipelago	Canary Archipelago	Madeira Archipelago
BIO3	BIO2	BIO2
BIO6	BIO12	BIO12
Artificial illumination	Euclidean distance from mixed agricultural lands	Artificial illumination
Euclidean distance from mixed agricultural lands		BIO6
Euclidean distance from hydrographical elements		Euclidean distance from mixed agricultural lands
		Euclidean distance from hydrographical elements

## Data Availability

The genetic data sources are presented in Table 1, while the occurrence data sources are indicated in Table 2, which includes published references. In cases where the occurrence data is unpublished, disclosure is withheld for conservation reasons. Requests for such data can be directed to the first author, S.T.

## Supplementary Materials

The following supporting information can be downloaded at: https://www.mdpi.com/article/10.3390/biology12070998/s1. Figures S1–S5: Response curves for the six variables used to model *Pipistrellus maderensis* potential distribution in Macaronesia; Variable importance estimates from *P.maderensis*’s SDM in Macaronesia region; Response curves for the five variables used to model *Pipistrellus maderensis* potential distribution in Madeira archipelago; Response curves for the five variables used to model *Pipistrellus maderensis* potential distribution in Azores archipelago; Response curves for the three variables used to model *Pipistrellus maderensis* potential distribution in Canary archipelago Titles.
