# Supplementary material for: Unveiling the Potential Distribution of the Highly Threatened Madeira Pipistrelle (Pipistrellus maderensis): Do Different Evolutionary Significant Units Exist?
Source: Biology (Basel). 2023 Jul 13;12(7):998. doi: 10.3390/biology12070998 (PMC10376549; doi:10.3390/biology12070998)
Supplement: Supplementary file 1 [file biology-12-00998-s001.zip › biology-2495427-supplementary.pdf]

Supplementary materials

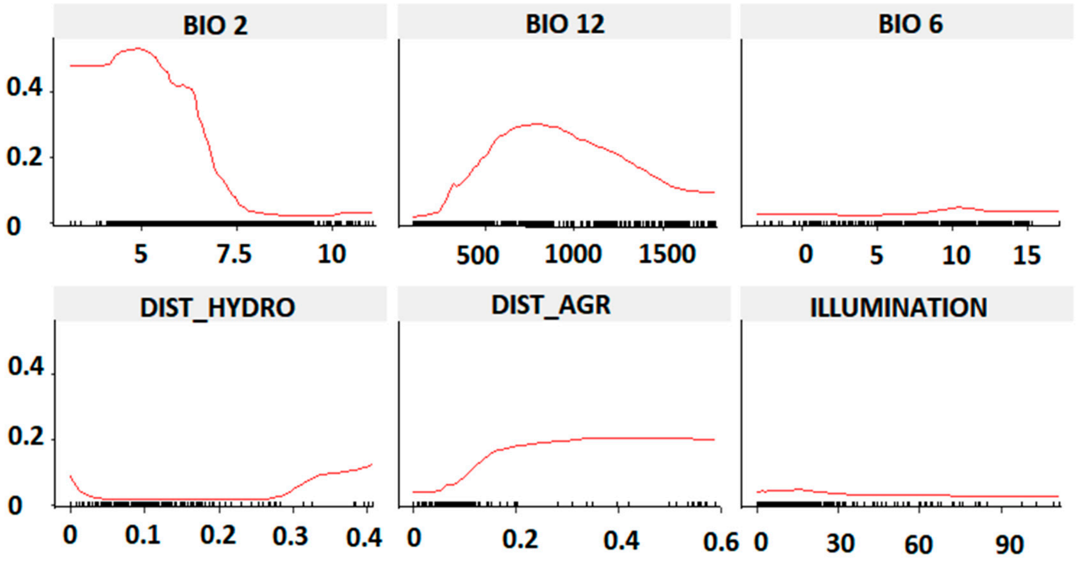

Figure S1. Response curves for the six variables used to model *Pipistrellus maderensis* potential distribution in Macaronesia obtained with the "biomod2" computer platform. Response curves correlate the probability of occurrence (y-axis) with values of the explanatory variables (x-axis). Each curve represents one variable (BIO 2 = mean diurnal range of temperature; BIO 12 = annual precipitation; BIO6 = mean temperature of the coldest month; DIST_HYDRO = Euclidean distance from hydrographical elements; DIST_AGR = Euclidean distance from agricultural areas; ILLUMINATION = artificial illumination. Distances are expressed in decimal degrees, precipitation in mm/%, temperature in °C and artificial illumination in 10^{-9} W /cm2 sr.

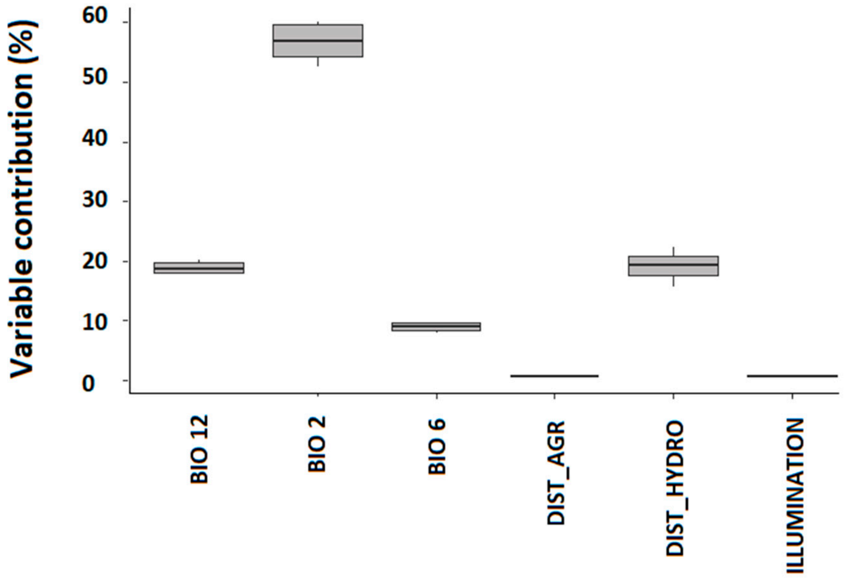

Figure S2. Variable importance estimates from *P.maderensis*'s SDM calibrated on all records from the Macaronesia region.

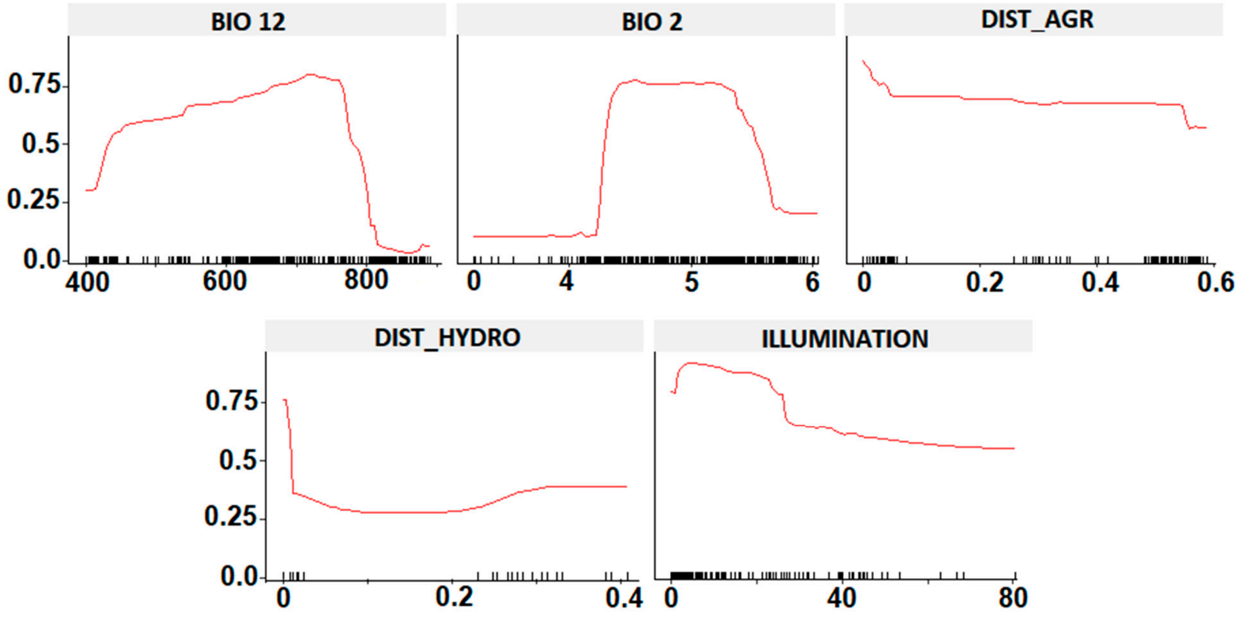

Figure S3. Response curves for the five variables used to model *Pipistrellus maderensis* potential distribution in the Madeira archipelago. Response curves correlate the probability of occurrence (y-axis) with values of the explanatory variables (x-axis). Each curve represents one variable (BIO 2 = mean diurnal range of temperature; BIO 12 = annual precipitation; DIST_HYDRO = Euclidean distance from hydrographical elements; DIST_AGR = Euclidean distance from agricultural areas; ILLUMINATION = artificial illumination. Distances are expressed in decimal degrees, precipitation in mm/%, temperature in °C and artificial illumination in 10^{-9} W /cm2 sr.

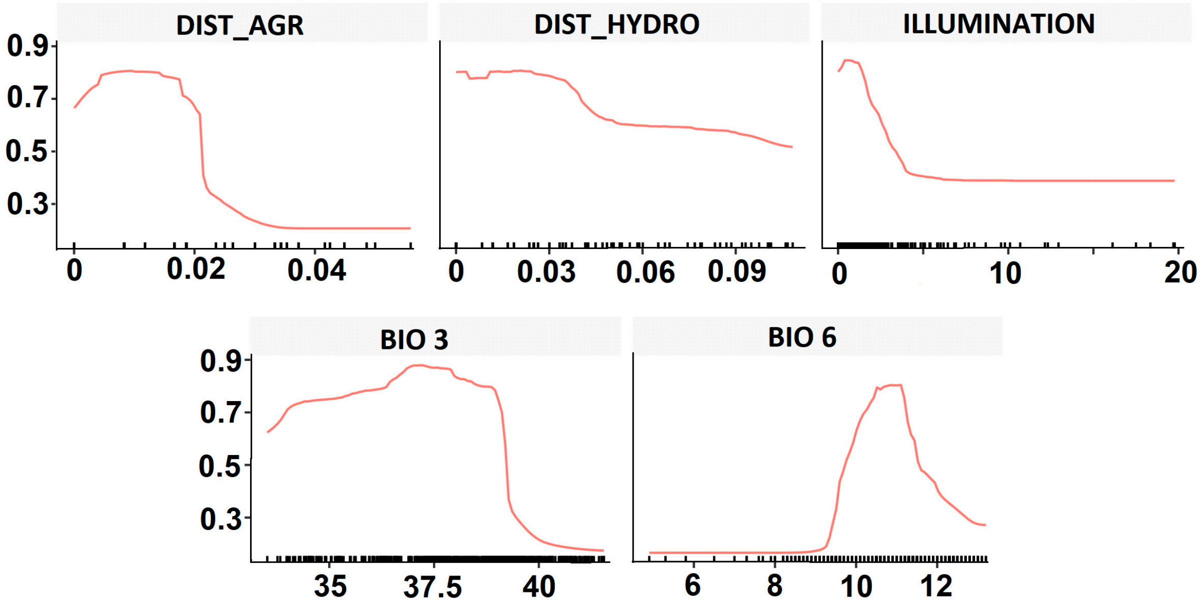

Figure S4. Response curves for the five variables used to model *Pipistrellus maderensis* potential distribution in the Azores archipelago. Response curves correlate the probability of occurrence (y-axis) with values of the explanatory variables (x-axis). Each curve represents one variable (BIO 3 = isothermality; BIO 6 = minimum temperature of the coldest month; DIST_HYDRO = Euclidean distance from hydrographical elements; DIST_AGR = Euclidean distance from agricultural areas; ILLUMINATION = artificial illumination. Distances

are expressed in decimal degrees, precipitation in mm/%, temperature in °C and artificial illumination in 10^{-9} W /cm2 sr.

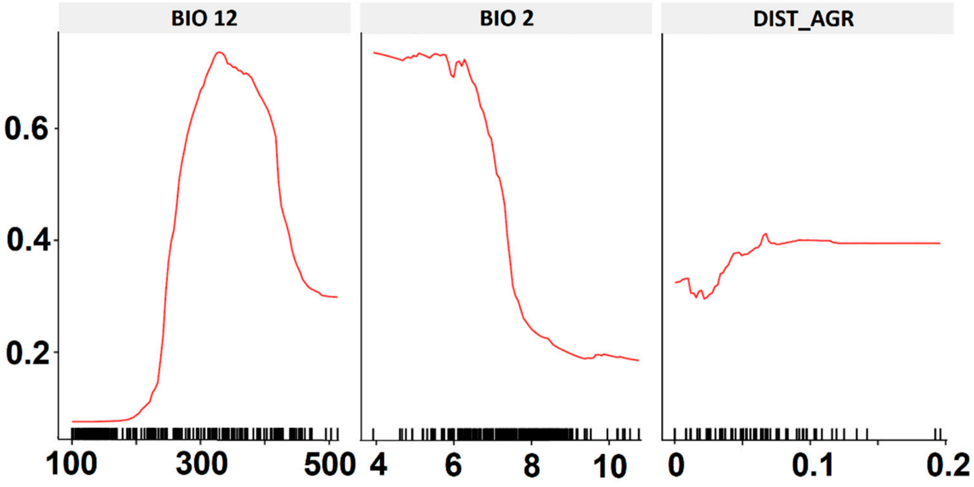

Figure S5. Response curves for the three variables used to model *Pipistrellus maderensis* potential distribution in the Canary archipelago. Response curves correlate the probability of occurrence (y-axis) with values of the explanatory variables (x-axis). Each curve represents one variable (BIO 2 = mean diurnal range of temperature; BIO 12 = annual precipitation; DIST_AGR = Euclidean distance from agricultural areas. Distances are expressed in decimal degrees, precipitation in mm/%, and temperature in °C.
